# Comprehensive Overview of Gastric Cancer Immunohistochemistry: Key Biomarkers, Advanced Detection Methods, and Perspectives

**DOI:** 10.3390/medicina62040683

**Published:** 2026-04-03

**Authors:** Bogdan Oprea

**Affiliations:** Department 1–Histology, Faculty of Medicine, University of Medicine and Pharmacy of Craiova, Petru Rares, 2, 200349 Craiova, Romania; bogdan.oprea@umfcv.ro

**Keywords:** immunohistochemistry, gastric cancer, biomarkers, advantages and limitations, perspectives

## Abstract

*Background and Objectives*: Immunohistochemistry (IHC) is a keystone in gastric cancer (GC) management, allowing treatment customization, including for advanced or metastatic diseases. This review aims to evaluate the critical role of IHC markers, analyzing their efficiency in molecular subclassification and prediction of response to gastric cancer-targeted therapies, while also describing state-of-the-art IHC techniques and perspectives. *Results*: The major challenges for the GC management were structured in two main sections, as follows: (i) the current paradigm of gastric neoplasia diagnosis, which includes subsections related to the methodological and morphological foundations, the epidemiological dynamics, and risk factors, as well as differential diagnosis of poorly differentiated tumors; and (ii) the progress in 3,3′-diaminobenzidine (DAB) application and advanced reagents in gastric cancer immunohistochemistry. *Discussion*: Considering the role of IHC and DAB, the following topics were successively addressed in seven sections: GC key biomarkers, such as human epidermal growth factor receptor 2 (HER2), programmed death-ligand 1 (PD-L1), and DNA replication mismatch repair (MMR) system, allow direct correlation between tissue morphology and protein expression; intestinal and gastrointestinal differentiation markers; emerging and aggressive histological subtypes; epithelial–mesenchymal transition, E-cadherin, and the process of tumor budding; implementation of innovative procedures in gastric cancer immunohistochemistry; and automation, quality control, and sustainability in the pathology laboratory. *Perspectives*: The main directions were focused on the integration of artificial intelligence (AI) algorithms for digital quantification of the IHC signal and also on the expansion of panels to new targets, such as Claudin 18.2 (CLDN 18.2), which redefines treatment approaches in advanced stages. *Conclusions*: Although faced with technical and biological limitations, immunohistochemistry remains indispensable in modern gastric oncology. The evolution towards digital pathology and the refinement of scoring criteria will transform IHC from a complementary test into a visual tool that is essential for personalizing oncological treatment.

## 1. Introduction

Gastric cancer represents a major public health challenge, representing the fifth most common form of cancer and the fourth highest cause of cancer death globally, despite advances in prevention and screening strategies [1,2,3,4,5]. Its uneven geographical distribution, multifactorial etiology, and pronounced biological complexity significantly complicate diagnosis and treatment [5].

In this context, IHC has established itself as the fundamental technique that bridges classical surgical pathology and modern molecular biology, providing critical information about protein expression directly in the architectural context of the tumor [6,7,8,9,10,11].

The evolution of the management of this pathology has undergone a profound transformation, moving from an approach based exclusively on the morphology visible in standard hematoxylin and eosin (H&E) staining to a molecular characterization, intended to guide towards personalized management. Therefore, the analysis of gastric malignant lesions advanced from purely morphological assessment to an integrative approach that includes detailed molecular and immunohistochemical profiling [11]. GC tumoral variety directly influences the response to therapy and the prognosis of patients [9,10,11,12,13,14].

Immunohistochemistry uses the specificity of antibodies to identify specific antigens in tissue sections [15,16], allowing the visualization of biomarkers that dictate not only prognosis but also eligibility for innovative treatments, such as immune checkpoint inhibitors [17,18] or anti-HER2 therapies [19].

Thus, the tissue origins of diseases can be identified in order to understand the biological behavior of tumors and to support complex clinical decisions. The role of IHC is amplified by the fact that many gastric cancers are diagnosed at advanced stages, where accurate characterization of the molecular profile on small endoscopic biopsy samples is vital for patient survival.

The use of immunohistochemical techniques on formalin-fixed, paraffin-embedded tissue sections has become an effective standard in surgical pathology, providing an essential bridge between gross pathology and precision medicine [9,11].

One of the most important aspects of modern gastric cancer diagnostics is the assessment of biomarker status to guide targeted therapies [20,21]. Biomarkers assessed by IHC, such as HER2, MMR, and PD-L1, are now indispensable tools for the comprehensive pathological evaluation of gastrointestinal malignancies [22,23,24,25].

Numerous studies have emphasized the importance of identifying biomarkers that can be predictive, indicating the likelihood of response to a specific therapy, providing information about the likely course of the disease [9,11,26,27,28,29,30,31,32,33]. For example, determination of HER2 status is mandatory in gastric cancer, although its correlation with prognosis remains a subject of debate in the specialist literature. Also, the assessment of PD-L1 using the combined positive score (CPS) requires the precise identification of lymphocytes and macrophages in the vicinity of tumor nests.

In addition, new markers such as CLDN 18.2 are entering current clinical practice for the selection of patients who may benefit from anti-CLDN18.2 therapies, placing the pathologist in a central role in the therapeutic decision-making process [12].

The heterogeneity of gastric cancer is not only morphological, but also molecular. The classifications proposed by The Cancer Genome Atlas (TCGA) and the Asian Cancer Research Group (ACRG) have provided new insights into how immunohistochemical markers can be used to subclassify patients into groups with distinct biological behavior [34,35,36]. The use of predictive markers has transformed the prognosis of patients with advanced or metastatic disease, where survival with conventional chemotherapy was often less than one year.

The current standard of care for advanced gastric adenocarcinoma involves the use of fluoropyrimidine- and platinum-based chemotherapy, but the selection of additional biological agents depends strictly on the immunohistochemical profile [37,38,39].

The complexity of the gastric carcinogenesis process, often described by Correa’s cascade, involves a progression from chronic gastritis to atrophy, intestinal metaplasia, dysplasia, and finally invasive adenocarcinoma, each stage being marked by molecular changes detectable by immunohistochemical methods [40,41].

Morphologically and molecularly, GC is a heterogeneous, multifactorial disease influenced by both genetic and environmental factors [42]. Thus, one of the most significant advantages of IHC is the ability to assess heterogeneity from inside the tumor in relation to the stroma and infiltrating immune cells.

Nowadays, the diagnosis of gastric neoplasia is no longer just the confirmation of the presence of a tumor mass, but a multidimensional process that includes high-resolution imaging staging, advanced endoscopic characterization assisted by artificial intelligence, and predictive biomarker profiling [43,44,45,46,47,48,49].

This emerging paradigm is essential, as gastric cancer is often diagnosed at advanced stages, where conventional therapeutic options offer limited benefit [49,50,51].

The use of DAB remains the cornerstone of these investigations, but advances in signal amplification systems, the use of nanozymes, and the integration of artificial intelligence have extended the capabilities of this classic reagent to new frontiers of sensitivity and multiplexing [52].

Tissue-destructive molecular techniques such as polymerase chain reaction (PCR) can dilute the signal of proteins expressed only by a small subpopulation of tumor cells, leading to falsely low results [53], while IHC allows direct visualization of these outbreaks. Concurrent testing increases sensitivity and improves patient response to biomarker-guided therapy.

The current review aims to analyze the role of immunohistochemical techniques in the management of gastric cancer, highlighting the advantages, limitations, and clinical perspectives in the context of precision medicine.

## 2. Materials and Methods

**Figure 1:**
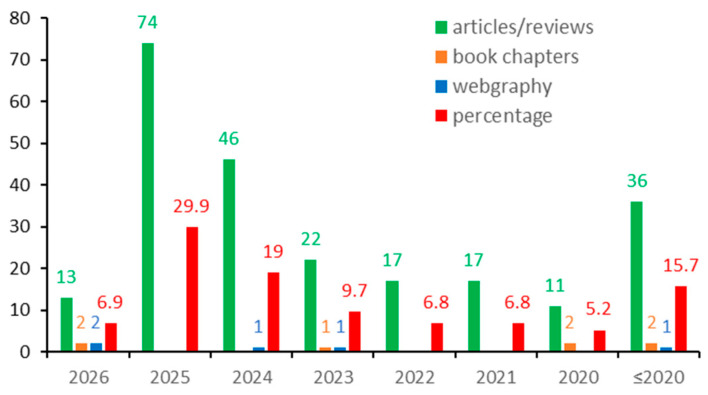
The number and percentage of bibliographical sources used in the current work distributed by years.

This review was developed based on systematized literature data from 248 bibliographic sources consisting of articles/reviews (236), book chapters (7), and webpages (5). Of the 236 articles/reviews, 224 (94.9%) were published in ISI (Web of Science) journals. The distribution of references by years is shown in Figure 1. It can be seen that 84.3% (209 references) are sources from the period 2020–2026, 55.8% from the last two years, and 15.7% (39 references) represent publications from years before 2020.

The Figures were created in Word or Excel by inserting Shapes, SmartArt, or Charts. The images were collected following the standard procedure and acquired using an Eclipse 90i (Nikon, Tokio, Japan) equipped with a QImaging Rolera cooled CCD camera microscope (Surrey, BC, Canada) with the following characteristics: eyepiece magnification power: 10×; lens magnification power: 20×. Google Gemini was partially used to assist in study design and when collecting certain data.

## 3. Results

### 3.1. Current Paradigm of Gastric Neoplasia Diagnosis

#### 3.1.1. Methodological Foundations and IHC Technical Mechanisms

The effectiveness of immunohistochemical studies in the detection of gastric malignant lesions depends on a sequence of rigorously controlled biochemical processes.

**Figure 2:**
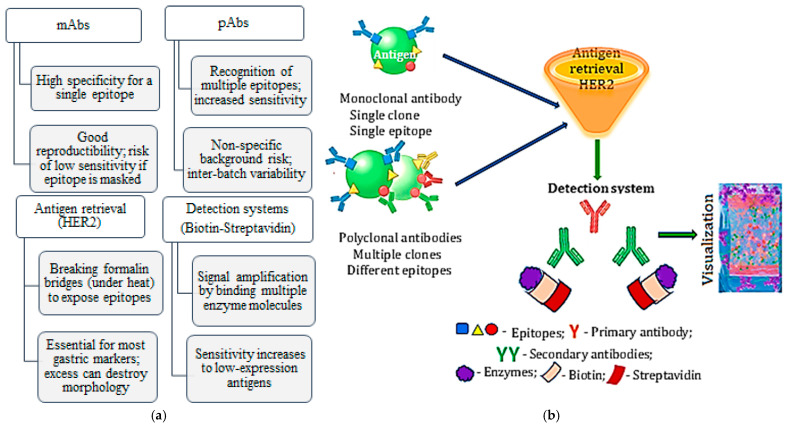
IHC core methodological components, role in gastric diagnosis, and impact on the outcome (**a**); synthetic succession of basic methodological components: antibody binding to an epitope; antigen retrieval; detection system; and visualization (**b**).

The technique is based on the binding of a monoclonal (mAbs) or polyclonal (pAbs) antibody to a specific epitope of a target protein. This interaction is subsequently visualized by a detection system that usually uses an enzyme, such as horseradish peroxidase (HRP), which catalyzes the transformation of a chromogenic substrate into a colored, insoluble product at the reaction site. Figure 2 systematizes the IHC basic methodological components, their role in gastric diagnosis, and the impact on the results (Figure 2a), as well as their systematic sequences until visualization (Figure 2b).

In tissue analysis, the mechanism of action of pAbs is often preferred due to their ability to generate a much stronger signal. As multiple antibody molecules bind to the same target protein, the intensity of the fluorescent or colorimetric signal is naturally multiplied. However, pAbs can produce higher “background noise” due to cross-reactions with similar proteins.

However, mAbs are the gold standard for precise diagnostics, such as the identification of specific tumor markers (e.g., HER2). They provide a much “cleaner” image, with minimal background noise, allowing for an unambiguous interpretation of the presence or absence of a specific molecular target. Thus, the choice between mAbs and pAbs in the laboratory is dictated by the balance between sensitivity (ability to detect small amounts) and specificity (ability to avoid false signals).

Monoclonal antibodies, recognizing a single epitope, have a limited mechanism of action in forming networks. Because all antibody molecules “compete” for the same spot on the antigen surface, they cannot create bridges between molecules unless the antigen possesses multiple identical copies of the same epitope (as in the case of repetitive polymeric structures). In most globular proteins, mAbs form only triplets (antigen–antibody–antigen) that are too small to precipitate or be detected by classical nephelometry. For this reason, agglutination and precipitation assays remain the predilection domain of polyclonal antibodies.

The mechanism of lattice formation is based on the bivalence of antibodies and the multivalence of antigens. When a bivalent polyclonal antibody binds two different epitopes on two different antigen molecules, it creates a “bridge”. The extension of this process leads to the formation of large three-dimensional complexes that precipitate from solution. Unlike cell lysis techniques such as reverse transcription-polymerase chain reaction (RT-PCR) or Western blotting (immunoblotting), IHC preserves the morphological integrity of the sample, which allows the pathologist to observe whether a protein is expressed in the nucleus, cytoplasm, or on the cell membrane.

This subcellular localization is often the key to diagnosis; for example, membrane staining is crucial for the assessment of the HER2 receptor, while nuclear staining is necessary for the assessment of proliferation markers such as Ki-67 or MMR. Implementing IHC in the pathology laboratory workflow brings advantages that go beyond the limits of simple morphological analysis, providing a functional dimension to histopathological diagnosis [54].

#### 3.1.2. Morphological Foundations and Oncogenic Cascades

Understanding the mechanisms underlying the malignant transformation of the gastric epithelium requires an analysis of the precancerous conditions identified in the literature.

Gastric and esophageal–gastric junction adenocarcinomas arise through a multistage process. The Correa cascade represents the classic model for intestinal-type adenocarcinoma [40,55,56], where environmental factors, especially *Helicobacter pylori* (*H. pylori*) infection, diet, and lifestyle, interact with the host’s genetic predisposition [57,58,59].

The morphological features and relevant IHC markers for the different stages of the Correa cascade are presented in Table 1 [40,41,56].

In the pathological context, IHC markers act as ancillary tests and not as independent diagnostic tools, providing additional information regarding tumor classification, cell lineage differentiation, proliferative activity, and prognosis. They must be interpreted in conjunction with standard histomorphological hematoxylin and eosin (H&E) staining.

Immunohistochemistry is used to monitor changes in protein expression during these transitions. For example, inactivation of the CDH1 gene, which encodes E-cadherin, is a critical event in the development of diffuse gastric cancer. The absence or abnormal expression of E-cadherin leads to loss of intercellular cohesion, allowing tumor cells to infiltrate the gastric wall as single cells or small clusters, often with a “signet ring” morphology [60]. The regulators for GC progression, such as Galectin-1, can regulate immune evasion and matrix remodeling [61].

As mentioned in the Introduction, the TCGA and ACRG proposed subclassifying patients into groups with distinct biological behavior [34,35,36,62]. These subgroups include Epstein–Barr virus (EBV)-positive tumors, tumors with microsatellite instability (MSI), tumors with chromosomal instability (CIN), and genomically stable (GS) tumors [63,64]. Many of these features can be assessed by IHC as an affordable surrogate for genomic sequencing.

#### 3.1.3. Epidemiological Dynamics and Redefinition of Risk Factors in 2026

Although the global incidence of gastric cancer has declined in recent decades in Western countries, the disease continues to represent a major public health problem, with over one million new cases reported annually worldwide [2,65,66]. Recent epidemiological observations highlight a shift in the anatomical distribution of tumors, with an increase in adenocarcinomas of the cardia and esophagogastric junction, in parallel with a decrease in distal forms traditionally associated with chronic infection [67,68]. An alarming phenomenon is the increase in the incidence of early-onset gastric cancer (EOGC) in people under 50 years of age, a cohort that often presents sporadic, diffuse forms with a more aggressive biology and increased resistance to standard treatments [69].

Risk factors have diversified in the context of modern lifestyle. While *Helicobacter pylori* infection remains the main carcinogen responsible for triggering the cascade of precancerous changes (atrophy, intestinal metaplasia, and dysplasia), other factors such as obesity, gastroesophageal reflux disease, and consumption of ultra-processed foods have gained ground in the modern etiology [59,70]. The role of Epstein–Barr virus (EBV) has also been reassessed, being implicated in approximately 10% of gastric adenocarcinomas, defining a molecular subtype with unique immunological characteristics and a more favorable overall prognosis [71]. Table 2 systematizes the risk factors that can initiate and develop gastric cancer.

The clinical picture of gastric cancer remains one of the most insidious in oncology, with early symptoms either absent or confused with functional dyspepsia. By the time classic signs such as unintentional weight loss, iron deficiency anemia, or persistent abdominal pain appear, the disease is often at an advanced or metastatic stage. For this reason, high clinical suspicion is vital, especially in patients with known risk factors or family history [70,71,72,73,74,75,76,77].

Upper gastrointestinal white light endoscopy (WLE) is a diagnostic standard, but its effectiveness critically depends on the quality of the examination. The current protocol recommends a systematic examination of all gastric segments (fundus, body, antrum, and pylorus) under adequate insufflation to avoid “blind spots” or blind areas [51,78].

A major barrier to early detection is the presence of mucus and debris, which is why pre-treatment with mucolytic and defoaming agents 30 min before the procedure has become a standard recommendation. Confirmation of the diagnosis requires taking a minimum of 6–8 biopsies from the edges and center of the lesion, as a single biopsy has a significant error rate due to tumor diversity and possible areas of peritumoral necrosis or inflammation [51].

#### 3.1.4. Differential Diagnosis of Poorly Differentiated Tumors

A recurrent diagnostic problem in digestive pathology is identifying the origin of a malignant tumor, especially when liver metastasis, colorectal carcinomatosis [79,80], or metastatic gastrointestinal adenocarcinoma disguised as a primary malignant bone tumor [81] are highlighted. The cytokeratin profile (CK7 and CK20) serves as a first-line phenotypic classifier.

Most gastric adenocarcinomas follow the CK7+/CK20- pattern, but there is considerable variability. Gastric intestinal-type adenocarcinoma may have a CK7-/CK20+ profile, making it almost impossible to distinguish morphologically and immunohistochemically from colorectal adenocarcinoma without the use of additional markers.

A recent case reported in the literature describes a gastric intestinal-type adenocarcinoma with a CK7-/CK20+ profile that metastasized to the urinary bladder, an extremely rare presentation that highlights the limitations of classical IHC panels [82].

Interpretation of key IHC markers related to the suspected tumor types is presented in Table 3.

The morphology of many gastric tumors can be ambiguous, especially in the case of “small cell” or “poorly differentiated” variants. IHC allows rapid differentiation between a gastric adenocarcinoma, a primary gastric lymphoma, and a gastrointestinal stromal tumor (GIST). The use of panels of antibodies directed to cytokeratin filaments (positive in carcinomas), lymphoid markers such as CD20 or CD30 (positive in lymphomas), and markers such as CD117 or DOG1 (positive in GIST) provides a certain diagnosis that dictates the clinical course [83,84,85,86,87,88].

When a patient presents with lymph node or liver metastases without an obvious primary tumor, IHC is the tool of choice to suggest the stomach as the origin. The marker CDX-2, a transcription factor involved in intestinal development, is often expressed in intestinal-type gastric adenocarcinomas [83]. Also, cytokeratin profiling (CK7 and CK20) can help to exclude other origins, such as lung or colon [83,91].

### 3.2. Progress in the DAB Application and Advanced Reagents in Gastric Cancer IHC

#### 3.2.1. DAB Evolution in the Context of Gastric Pathology

As described in the previous sections, the GC clinical management evolution was marked, in the last decade, by a paradigm change, passing from a uniform surgical and chemotherapeutic approach to a precision medicine based on detailed molecular profiling of each tumor. In this context, IHC has reasserted itself as the fundamental diagnostic tool, serving not only for morphological classification but also for the identification of specific therapeutic targets that can significantly prolong the survival of patients with advanced disease [7,91,92,93]. Due to the GC tumor nonuniformity, the use of highly sensitive and reproducible imaging techniques is required.

DAB is the standard chromogen in immunohistochemistry due to its physicochemical properties that allow clear and permanent visualization of target antigens under the light microscope [94,95]. The staining reaction is mediated by the HRP, which catalyzes the oxidation of DAB in the presence of hydrogen peroxide (H_2_O_2_) [96]. The biochemical process involves the formation of a reactive radical intermediate that rapidly polymerizes to produce an insoluble brown deposit at the site of antibody–antigen interaction [97]. The general equation for the DAB oxidation reaction can be represented as follows:

$$HRP+H_{2}O_{2}+DAB\overset{to}{\to }{DAB}^{•+}+H_{2}O+oxidized\;HRP$$

This brown polymer is extremely stable, being insoluble in water, alcohol, and organic solvents such as xylene, which facilitates permanent mounting of slides and their archiving for long periods without signal degradation [91,94]. In the diagnosis of gastric cancer, DAB stability is crucial, given that samples may be subject to subsequent re-evaluation for new biomarkers as therapeutic options evolve [91].

**Figure 3:**
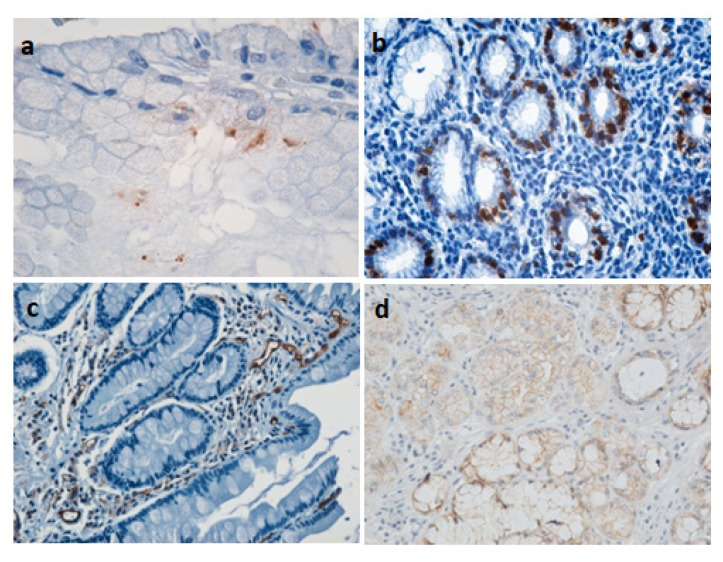
Visualizing protein expression for suspected gastric cancer or premalignant lesions; (**a**)—*Helicobacter pylori*; (**b**)—PCNA tumor marker; (**c**)—CD34 and f(VIII) cocktail; (**d**)—membrane adhesion staining using E-cadherin (DABx200).

Figure 3 shows specific DAB stains for suspected gastric cancer or premalignant lesions evaluated in the presence of specific markers.

*H. pylori* is responsible for 74.7–89.0% of gastric cancer cases; the bacterium colonizes the gastric epithelium, causing chronic inflammation, oxidative stress, DNA damage, and changes in stomach cells from chronic gastritis and atrophy to cancer [98].

Proliferating Cell Nuclear Antigen (PCNA) is used in histopathology to assess the rate of tumor cell proliferation. A high PCNA index often correlates with aggressive, invasive, or high-grade tumors. PCNA provides information about the biological behavior of gastric cancer and its degree of aggressiveness [99].

Increased expression of vascular endothelial growth factor (VEGF) and CD34 (microvascular density marker) in gastric cancer indicates intense angiogenesis [100], significantly correlated with advanced tumor stage, lymphatic invasion, metastasis, and increased risk of perioperative hemorrhage. Their co-expression serves as a negative prognostic indicator and biomarker for risk assessment. Patients with high levels of VEGF and CD34 have a poorer prognosis and a higher risk of bleeding during surgery [101,102].

E-cadherin is a useful marker in diagnosis and prognostic assessment. Decreased expression occurs with mutations in the CDH1 gene, epigenetic (methylation) changes, or *H. pylori* infection. Reduced E-cadherin staining is correlated with higher grades of malignancy, deeper invasion, and poorer patient survival. E-cadherin maintains normal epithelial structure. When membrane staining disappears, cells become motile, losing their cohesion [103].

#### 3.2.2. Optimization and Modern DAB Generations

**Figure 4:**
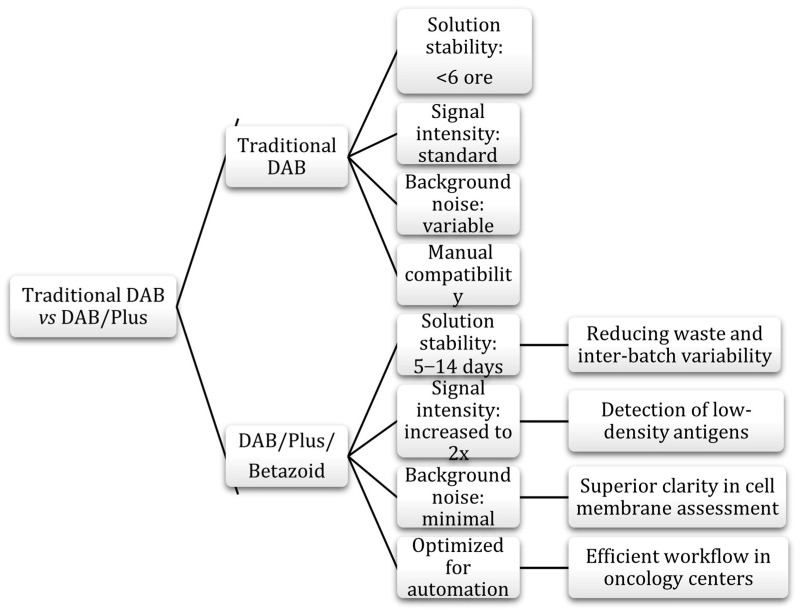
DAB traditional versus DAB/Plus.

Over the years, DAB formulations were optimized to overcome the sensitivity and stability limitations of hand-prepared solutions [94]. Third-generation products, such as Betazoid DAB, have been developed to provide superior staining intensity and working solution stability of up to several days or even weeks, as opposed to the few hours typical of traditional formulations [94,104]. Figure 4 shows a comparison of the characteristics of traditional DAB and DAB/Plus (solution stability, signal intensity, background noise, and compatibility), highlighting the advantages of the latter.

A major advance in gastric cancer immunohistochemistry has been the transition from avidin–biotin complex (ABC)-based methods to synthetic polymer-based detection systems [105]. Gastric tissue, being metabolically active, often contains high levels of endogenous biotin, which can generate false-positive signals when using biotin-based detection systems [104,105].

Modern polymeric systems, UltraView (Ventana), OptiView (Ventana), and EnVision (Dako/Agilent) use a polymeric backbone to which multiple HRP molecules and secondary antibodies are directly attached [106]. This configuration not only eliminates endogenous biotin interference but also provides intrinsic signal amplification. The polymeric structure allows for the deposition of a larger amount of DAB at the binding site of the primary antibody, increasing the signal-to-noise ratio. In addition, the use of micro-polymers or Fab (fragment antigen-binding) antibody fragments reduces the size of the detection complex, facilitating better penetration into dense or fibrotic tissues, often characteristic of diffuse gastric adenocarcinoma [7,107].

## 4. Discussion

### 4.1. Precision Biomarkers in Gastric Cancer: The Role of IHC and DAB

One of the most important aspects of modern gastric cancer diagnosis is the assessment of biomarker status guiding targeted therapies [108,109,110,111,112,113,114,115,116,117].

The process begins with adequate sample collection, where the number of biopsies and tumor representativeness play a critical role, given the intratumoral complexity of protein expression [23,111,112,113].

#### 4.1.1. Human Epidermal Growth Factor Receptor 2 (HER2)

HER2 is overexpressed in approximately 22% of esophagogastric junction cancers and 15–20% of distal gastric cancers. HER2 testing by IHC has become a basic criterion for patient selection following the landmark ToGA trial, which demonstrated a clear survival benefit with the addition of Trastuzumab to standard chemotherapy [112]. The HER2 testing algorithm is presented systematically in Table 4 [54,118,119,120].

Thus, assessment of HER2 status is essential for selecting patients eligible for treatment with Trastuzumab in combination with chemotherapy or for new antibody–drug conjugates (ADCs) such as Trastuzumab deruxtecan (T-DXd) [115,116,117]. The use of IHC for HER2 offers a major economic advantage, as a rapid screening method, that allows the more expensive and time-consuming fluorescence in situ hybridization (FISH) technique to be reserved only for the 2+ score segment [113].

##### Challenges of HER2 Assessment: From Heterogeneity to Score Discrepancies

*1. Heterogeneity and Membrane Staining Pattern*

IHC particularities in gastric cancer compared to breast cancer include the incomplete nature of membrane staining. In the stomach, tumor cells often show a “basolateral” or “U”-shaped lateral staining, which should be considered positive [114,115], in contrast to the requirement for complete circumferential staining in breast cancer [118]. The HER2 receptor, encoded by the ERBB2 gene, is a proto-oncogene located on chromosome 17q21 [119]. Amplification of this gene leads to overexpression of the HER2 protein on the membrane of tumor cells, which triggers intracellular signaling cascades that promote cell proliferation and tumorigenesis.

Heterogeneity of HER2 expression is much more common in gastric cancer (in up to 79.3% of cases) than in breast cancer (approximately 5%) [118,121], manifesting by the presence of distinct tumor clones, with different levels of gene amplification or protein overexpression, unevenly distributed in the tumor mass. This biological feature induces a major risk of sampling error in endoscopic biopsies. A biopsy fragment may come from a HER2-negative area of an overall positive tumor, resulting in a false negative [121]. Studies indicate that the “positive conversion” rate (negative biopsy but positive resection) can reach 34.3% [121]. To minimize this risk, it is recommended to collect at least four to six viable tumor fragments [122]. When the number of fragments is more than four, the predictive capacity of HER2 status decreases drastically, generating discrepancies in approximately 12.3% of paired cases [118].

*2. Technical Vulnerabilities: Antibody Clones and Detection Systems*

IHC performance is highly dependent on the affinity and specificity of the antibody clone used. Although HercepTest (Dako) was used in the ToGA study, other clones such as 4B5 (Roche/Ventana) or A0485 have demonstrated superior sensitivity in detecting low levels of protein [42,123,124]. The historically used clone CB11 has been found to be insufficiently sensitive for use as a first-line screening test, exhibiting a high false-negative rate compared to fluorescence in situ hybridization (FISH) [123].

Interpretation may also be compromised by nonspecific reactivity of benign gastric epithelium or intense cytoplasmic staining, which may mask the authentic membrane signal [125]. In cases of signet ring cell carcinoma (SRCC), the unique cellular morphology, with abundant mucin-filled cytoplasm pushing the nucleus to the periphery, may lead to artifactual staining that mimics membrane overexpression, causing false-positive results [125].

*3. Scoring limitations*

Although HER2 is a well-established marker in breast cancer, its application in gastric cancer presents unique challenges. GC complexity implies that areas of positivity can coexist with areas of negativity within the same tumor. For this reason, the scoring criteria have been adapted. In a resection specimen, a score of 3+ is defined by strong membranous staining (complete, basolateral, or lateral) in ≥10% of tumor cells. In biopsy specimens, due to tissue fragmentation, any group of at least five tumor cells with strong staining is considered sufficient for a score of 3+.

*4.* *Inter-laboratory variability*

Inter-laboratory variability remains a documented problem in the literature [126]. Thus, excellent agreement was established for clearly positive cases (score 3+) and very good for negative cases (score 0 or 1+), but much lower agreement for equivocal cases (score 2+). As shown in Table 4, cases with a score of 2+ by IHC must be confirmed by in situ hybridization (ISH), such as fluorescence in situ hybridization (FISH) or chromogenic in situ hybridization (CISH), to detect gene amplification.

##### Current Approaches

Recently, the concept of “HER2-low” (IHC score 1+ or 2+ without FISH amplification) has begun to be investigated as a distinct clinical subgroup [111,127]. Studies suggest that HER2-low patients present with intermediate clinicopathological features and may represent a target for novel ADCs [128,129]. The incidence of HER2-low is reported to be higher in biopsy specimens (approximately 34.9%) compared to resection specimens (21.0%), suggesting a potential underestimation of HER2 status [111].

The use of monoclonal antibodies such as 4B5 on automated platforms (Benchmark ULTRA) with UltraView DAB detection systems has demonstrated considerable sensitivity in whole-tissue sections [130,131]. However, accuracy depends on the use of imaging reagents that can clearly delineate the cell membrane. In equivocal cases (IHC 2+), FISH or Silver-enhanced In Situ Hybridization (SISH) is used to confirm gene amplification [131,132]. Studies indicate that although SISH also uses a bright-field silver deposition reaction, IHC remains the primary screening method because patients with ISH-positive but IHC-negative tumors do not always benefit from Trastuzumab therapy [118,131,133].

#### 4.1.2. Immune Checkpoints: Programmed Death-Ligand 1 (PD-L1) and CPS Scoring

The immunotherapy implementation in gastric cancer treatment has necessitated the evaluation of the expression of the programmed death-ligand 1 (PD-L1) protein by immunohistochemistry [21,34]. PD-L1, expressed on the surface of tumor cells or infiltrating immune cells, interacts with the PD-1 receptor on T lymphocytes, suppressing the antitumor immune response [110]. Blocking this interaction with checkpoint inhibitors allows the reactivation of the immune system [134].

In gastric cancer, unlike in lung cancer, where the Tumor Proportion Score (TPS) is used, the standard method of evaluation is the combined positive score (CPS). This score evaluates the ratio of all PD-L1-positive cells (tumor cells, lymphocytes, and macrophages) to the total number of viable tumor cells, multiplied by 100. Although the result can theoretically exceed 100, the maximum score reported is capped at 100. A specimen is considered suitable for evaluation only if it contains at least 100 viable tumor cells. The CPS is determined according to the following formula [135]:

$$CPS=\frac{No.\;of\;PD-L1\;positive\;cells\;(tumor\;cells,lymphocytes,macrophages)}{Total\;no.\;of\;viable\;tumor\;cells}\times 100$$

Cut-off values commonly used in clinical trials are CPS ≥ 1, CPS≥ 5, or CPS ≥ 10, depending on the specific drug and line of treatment [34,136,137]. For example, the addition of nivolumab to chemotherapy has demonstrated a significant survival benefit in patients with HER2-negative gastric adenocarcinoma and CPS ≥ 5. However, PD-L1 diversity is a major diagnostic barrier. PD-L1 expression has been observed to be often more frequent on infiltrating immune cells (IICs), namely, leukocytes, specifically lymphocytes, macrophages, neutrophils, and dendritic cells, than on tumor cells (TCs) [110,136,137].

Furthermore, the discordance rate between primary tumor and metastases can be as high as 32.4%, raising questions about the most representative specimen for testing. Studies show that PD-L1 positivity is correlated with an EBV-positive subtype [64,138,139] and MMR deficiency, providing a strong rationale for the use of immunotherapy in these specific groups [64,140].

##### Interpretive Pitfalls

The CPS methodology is extremely laborious and susceptible to significant interobserver variability. Identifying cells to be included or excluded from the CPS calculation is a common source of error. The pathologist should exclude tumor cells with exclusively cytoplasmic staining and immune cells in areas of necrosis, as well as benign epithelial cells or areas of intestinal metaplasia, even if they are positive.

Granulocytes (neutrophils, eosinophils) and plasma cells should also be excluded, as their inclusion may lead to a false-positive score.

The efficacy of chemotherapy in combination with immune checkpoint inhibitors (ICIs) is closely correlated with the CPS level [141]. Sources of error in CPS scoring are discussed below [42].

*Cell differentiation*: It is often difficult to distinguish between small, poorly differentiated tumor cells and peritumoral macrophages or lymphocytes, both of which may express PD-L1. Macrophages should be counted in the numerator, but granulocytes, plasma cells, and stromal cells (fibroblasts) should be excluded, a task that becomes extremely difficult in areas of dense inflammation.

*Accuracy of the denominator*: Estimating the total number of viable tumor cells is a visual approximation. In tumors with abundant desmoplastic stroma or high mucin content, pathologists may overestimate or underestimate this number, significantly altering the final result, especially around the critical thresholds of CPS ≥1, ≥5, or ≥10.

*Signal intensity and location*: The CPS standard requires counting any membrane staining intensity (partial or complete) for tumor cells and any membrane or cytoplasmic intensity for immune cells. Isolated cytoplasmic staining in tumor cells should be excluded, but in practice, it is often erroneously included.

##### Interobserver Variability and Inter-Assay Discordance

Concordance studies have shown that the agreement between pathologists for CPS is relatively “modest”, with intraclass correlation coefficients (ICCs) ranging from 0.45 to 0.57, even after specialized training sessions [142]. This variability is more pronounced in the case of biopsies than in the case of resection specimens, due to the limited volume of tissue and the frequent presence of artifacts [142].

Furthermore, the use of different assays (22C3, 28-8, and SP263) on different platforms can generate divergent results. Although the 22C3 (used for pembrolizumab) and 28-8 (used for nivolumab) assays show good overall correlation, they are not perfectly interchangeable [143]. The SP263 assay tends to be more “permissive”, often reporting higher CPSs than 22C3 for the same sample, which may influence patient eligibility for treatment with nivolumab or pembrolizumab depending on regional regulations [124,144].

#### 4.1.3. Mismatch Repair and Microsatellite Instability (MMR/MSI)

Testing the status of the mismatch repair (MMR) system by IHC is an essential component of the initial pathological evaluation. MMR deficiency (dMMR) results from the loss of expression of one or more MMR proteins (MLH1, MSH2, MSH6, and PMS2), leading to the accumulation of mutations in microsatellite regions of DNA and microsatellite instability (MSI) [145,146,147,148,149,150].

In gastric cancer, dMMR/MSI is present in approximately 4–24% of cases and is associated with certain distinctive clinicopathological features [151,152]. PPatients in this category tend to be older, female, and 22-48% have the tumors located in the distal portion of the stomach, the antrum. [153,154]. Morphologically, these tumors often show a dense peritumoral and intratumoral lymphocytic infiltrate (lymphoepithelioma-like gastric carcinoma: LELGC) [155].

The interpretation of IHC for MMR proteins is binary: (i) pMMR (proficient mismatch repair) profile, when the nuclear expression of all four proteins in tumor cells is maintained; and (ii) dMMR profile is characterized by complete loss of nuclear expression for at least one protein (stromal cells and lymphocytes serving as internal positive controls).

The clinical importance of MMR status is twofold: (i) from a prognostic perspective, patients with localized disease and dMMR have a better prognosis compared with those with pMMR; and (ii) from a predictive perspective, these tumors are generally resistant to standard perioperative chemotherapy, but are highly sensitive to immunotherapy due to the high tumor mutational load that generates numerous neoantigens [155,156,157,158].

Thus, MSI/dMMR status is a crucial agnostic biomarker for identifying patients with an exceptional response to immunotherapy. Approximately 10–20% of gastric adenocarcinomas exhibit this phenotype [153], which results from loss of function of the MLH1, MSH2, MSH6, or PMS2 proteins.

Immunohistochemistry is the preferred screening method due to its low cost, universal availability, and ability to identify the specific missing protein, which guides genetic testing for Lynch syndrome [159].

A major drawback of IHC in MMR testing is false-negative results for dMMR. These occur when a missense mutation results in the production of a catalytically inactive protein that retains its antigenic structure and is thus detected by the IHC antibody [160].

Recent studies suggest that Next-Generation Sequencing (NGS) is superior to IHC for detecting MMR deficiency, identifying a higher percentage of dMMR patients who were missed by immunohistochemistry [161]. However, in current practice, IHC remains the first-line recommendation according to the American Society of Clinical Oncology and the College of American Pathologists (ASCO/CAP) 2022–2023 guidelines, due to its rapidity (turnaround time of 2–5 days vs. 2–4 weeks for NGS).

#### 4.1.4. Claudin 18.2: A New Frontier in Gastric Pathology (CLDN18.2)

Claudin 18.2 has emerged as a highly successful therapeutic target. In normal gastric mucosa, CLDN18.2 is strictly localized in the tight junctions of differentiated epithelial cells, making it inaccessible to circulating antibodies. However, once cell polarity is lost during carcinogenesis, the protein becomes exposed across the entire surface of the cancer cell membrane [47,162,163].

Thus, there are important methodological challenges. CLDN18.2 expression shows significant unevenness inside the tumor, with lower intensities often observed in its center compared to the surface [163]. Furthermore, although there is a good overall concordance (over 70%) between the primary tumor and metastases (including peritoneal metastases), inhomogeneity can lead to false-negative results on small biopsies [163]. Interestingly, CLDN18.2 expression appears to be more frequent in diffuse-type adenocarcinoma and in tumors that do not overexpress HER2, providing a therapeutic window for this subgroup of patients [163].

The standard protocol uses the VENTANA CLDN18 (43-14A) RxDx Assay in combination with the OptiView DAB Detection System. The cut-off for positivity in clinical trials (SPOTLIGHT and GLOW) is ≥75% of tumor cells, with moderate-to-strong membrane staining intensity (≥2+). This requirement of “massive overexpression” ensures the stability and accuracy of the DAB reagent, which is essential to avoid underestimation of the percentage of positive cells, which would deprive patients of potentially life-saving treatment [164,165].

The phase III clinical trials SPOTLIGHT and GLOW used IHC to select patients for Zolbetuximab [165,166,167]. The prevalence of this biomarker is approximately 35–40% among HER2-negative advanced gastric adenocarcinomas. The fact that this marker tends to be more frequent in the diffuse (Lauren) type than in the intestinal type (48.9% vs. 38.9%) offers a vital therapeutic option for a subset of patients with a traditionally poor prognosis [165].

##### Specificity and Rigor in Scoring CLDN18.2

Technical challenges associated with CLDN18.2 include [12,168,169,170,171,172,173,174,175]: (i) clone specificity—use of clone 43-14A is essential, as other clones may show cross-reactivity or suboptimal sensitivity; (ii) staining artifacts—only linear membrane staining should be scored; (iii) cytoplasmic staining—although common in tumors with strong expression, it should not be considered; (iv) variability of metastases—significant discordances have been observed between primary tumor and nodal metastases, suggesting that testing for metastases may be necessary if the primary tumor is negative; and (v) intestinal metaplasia is a critical checkpoint—this often shows weak or moderate staining intensity, serving as an internal control system for staining quality, but may induce sampling errors in small endoscopic biopsies.

#### 4.1.5. Fibroblast Growth Factor Receptor 2b (FGFR2b)

Overexpression of the FGFR2b protein has been identified as a relevant biomarker in approximately 30–40% of gastric cancer cases, especially in diffuse subtypes [176]. Interestingly, protein overexpression detected by IHC with DAB is much more frequent than FGFR2 gene amplification detected by NGS or FISH (which occurs in only 3–10% of cases). This discrepancy highlights the importance of IHC as a screening tool for drugs such as Bemarituzumab, where determination of cell surface receptor density by brown staining is more predictive of therapeutic response than genomic status [177,178,179]. A systematic presentation of the biomarkers discussed above is given in Table 5.

### 4.2. Intestinal and Gastrointestinal Differentiation Markers: CDX2 and SATB2

To refine the differential diagnosis between gastric and colorectal tumors, pathologists use nuclear transcription factors such as CDX2 and SATB2 [79,80].

Caudal-type homeobox 2 (CDX2) is essential for intestinal differentiation and is expressed in approximately 95% of colorectal adenocarcinomas. However, CDX2 is also frequently positive in gastric adenocarcinoma, especially in areas with intestinal metaplasia or the intestinal Lauren subtype, which reduces its specificity for colorectal origin [79,80].

In this context, Special AT-rich sequence-binding protein 2 (SATB2) has been shown to be a much more specific marker. SATB2 is expressed in appendiceal and colorectal epithelium [79,80,193] but is almost always negative in gastric, pancreatic, and biliary adenocarcinomas. A panel composed of SATB2, CK20, and CDX2 offers high sensitivity and specificity [79,80,193] for confirming the colorectal origin of a liver metastasis [80]. The importance of SATB2 is also highlighted in the diagnosis of Krukenberg tumors (ovarian metastases from primary gastrointestinal tumors). Studies show that three-quarters of primary gastric tumors are positive for CDX2, but only extremely rare cases express SATB2. Thus, a CDX2+/SATB2- profile, in an ovarian tumor with “signet ring” cells, indicates the diagnosis of a gastric origin, while a SATB2+ profile supports a colorectal or appendiceal origin [193].

### 4.3. Emerging and Aggressive Histological Subtypes: GAED and GA-FG

Immunohistochemical analysis has allowed the identification of rare gastric cancer entities that require a specific clinical approach. Two such subtypes are gastric adenocarcinoma with enteroblastic differentiation (GAED) and gastric adenocarcinoma of the fundic gland (GA-FG) [194,195].

GAED is a highly aggressive variant characterized by the production of oncofetal markers. Histologically, these tumors present large cells with clear cytoplasm, organized in tubules or cords, resembling the fetal intestine. The typical IHC profile includes [194]: (a) Glypican-3 (GPC3)—expressed in 83% of cases; (b) spalt-like (SALL4) family of 4 C_2_H_2_ zinc finger transcription factors—expressed in 72% of cases; and (c) alpha-fetoprotein (AFP)—expressed in 45% of cases. In contrast to conventional gastric adenocarcinoma, GAED has a much higher rate of liver metastasis (31% vs. 6%) and extensive lymphatic/venous invasion, which dictates a much worse prognosis [194].

On the other hand, GA-FG represents a variant with a much more indolent behavior. These tumors mimic the architecture of normal fundic glands and are predominantly composed of cells that resemble chief cells or parietal cells. The diagnosis is confirmed by [195]: Pepsinogen-I—Specific marker for fundic chief cells; and MUC6—Expressed in fundic and pyloric glands.

Most cases of GA-FG are diagnosed at an early stage (invasion limited to the mucosa or submucosa) and rarely present with nodal metastases, being often treatable by endoscopic resection [195].

### 4.4. Epithelial–Mesenchymal Transition, E-Cadherin, and the Process of Tumor Budding

The invasive and metastatic capacity of gastric cancer is closely linked to the process of epithelial–mesenchymal transition (EMT). During EMT, epithelial cells lose their polarity and intercellular junctions, acquiring a mobile mesenchymal phenotype [196]. Loss of membrane expression of E-cadherin is the best-known IHC marker of this process [197]. E-cadherin is a transmembrane glycoprotein that maintains epithelial integrity by binding to adjacent cells. In gastric cancer, its reduction or absence is correlated with high histological grade, the presence of nodal metastases, and an advanced stage of the disease [197].

**Figure 5:**
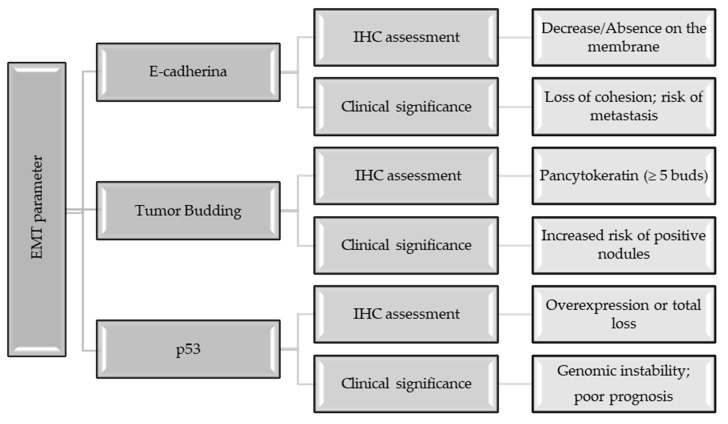
IHC evaluation and clinical significance for EMT parameters.

Another morphological marker of EMT is tumor budding (TB), defined by the presence of isolated tumor cells or groups of ≤4 cells at the invasion front. Assessment of tumor budding by IHC (using pancytokeratin staining to highlight small cell buds hidden in the inflammatory stroma) is an independent predictor of nodal metastasis [197]. In GC, alteration of the p53 protein, either by overexpression (missense mutation) or total loss of function, contributes substantially to setting off EMT, genomic instability, and poor clinical prognosis [198,199,200]. The information is revealed in a concise manner in Figure 5.

The correlation between high tumor budding and suppression of E-cadherin immunoexpression indicates an increased aggressive potential, with tumor cells being able to easily separate from the primary tumor and migrate through the peritumoral stroma towards lymphatic and blood vessels [201,202].

### 4.5. Implementation of Innovative Procedures in Gastric Cancer Immunohistochemistry

#### 4.5.1. Synthetic Conclusions Regarding the IHC Limits

Gastric cancer requires increasingly refined diagnostic methods to identify patients who may benefit from targeted therapies and immunotherapy. IHC is the cornerstone of this selection process, but traditional techniques have certain limitations related to tumor inconsistency, subjectivity of interpretation, and interobserver variability. GC exhibits one of the highest levels of complexity among all solid tumors, manifested by significant variations in protein expression between different regions of the same tumor (intratumoral) or between the primary tumor and metastatic sites (intertumoral).

This problem is exacerbated in the case of PD-L1 scoring using the CPS algorithm, which requires accurate counting of positive tumor cells, lymphocytes, and macrophages relative to total viable tumor cells. Discrepancies frequently occur at clinical decision thresholds, such as CPS ≥ 1, ≥5, or ≥10, where a small difference in interpretation can change a patient’s eligibility for immune checkpoint inhibitors.

At the same time, pre-analytical variables and antigenic integrity can induce a distortion of IHC results. Their quality is largely determined by the steps taken before staining [203]. Prolonged cold ischemia (time from sampling to fixation) and the duration of fixation in 10% neutral buffered formalin are critical. Current guidelines recommend a minimum of 6 h and a maximum of 72 h of fixation; under-fixation leads to edge artifacts, while over-fixation masks epitopes through excessive cross-linking, requiring aggressive antigen retrieval methods that can degrade tissue morphology [203].

#### 4.5.2. Advanced Methods in Gastric Cancer Immunohistochemistry

Implementation of advanced techniques offers efficient solutions to overcome limitations, ensuring superior accuracy and critical reproducibility in the case of personalized oncology. In this regard, certain strategies are revealed, as follows: (1) advanced signal amplification strategies, such as TSA (Tyramide Signal Amplification) and hybridization chain reaction (HCR); (2) multiplex immunohistochemistry (mIHC); (3) artificial intelligence (AI)-assisted digital pathology; and (4) spatial proteomics techniques.

##### Advanced Signal Amplification Strategies: TSA and HCR

For the detection of extremely low-abundance molecular targets, such as certain immune checkpoints or RNA transcripts, the standard sensitivity of DAB can be limited. Two major technologies have been developed to enhance the IHC signal, namely TSA and HCR.

TSA or catalyzed reporter deposition (CARD) technology uses peroxidase activity to deposit tyramide molecules conjugated to a marker (fluorophore or biotin) around the antibody binding site [204,205]. This process generates a signal amplification of up to 100–1000-fold without increasing background noise. Because DAB is opaque and cannot be easily used in multiplexing, TSA allows the sequential use of DAB or other chromogens in multiplexing procedures. In gastric cancer, TSA is a core technology multiplex mIHC [206], or in multiplex immunofluorescence (mIF) [206,207], to map the tumor microenvironment (TME) [207], such as simultaneous mapping of tumor cells (e.g., pancytokeratin) and various subpopulations of T lymphocytes or cytotoxic T cells (CD8+) and macrophages. In this way, a detailed picture of immune interactions is provided in the tumor stroma [208] or, as studies from 2026 show, the efficiency of detecting circulating tumor cells (CTCs) can be improved [209].

Consequently, in the GC context, TSA allows [207,208,209]: (a) the detection of rare proteins by visualizing cytokine receptors or tumor stem cell markers that are expressed at levels below the detection threshold of classical IHC; (b) reduction in reagent consumption by using dilutions of primary antibodies, diminishing costs and background noise; (c) immuno-detection without cross-reactivity, because tyramide is covalently bound, and antibodies can be thermally eluted; and (d) the successive use of antibodies from the same host species in complex panels.

HCR represents a recent advance that combines the specificity of hybridization probes with the amplifying power of DNA self-assembly. In contrast to traditional enzymatic methods, HCR v3.0 (third-generation hybridization chain reaction) technology utilizes DNA “hairpins” that open and polymerize only in the presence of the target probe [210,211]. State-of-the-art versions of HCR-Cat (hybridization chain reaction with catalyzed amplification) integrate HRP activity to allow the deposition of DAB or fluorescent chromogens, achieving ultrasensitive detection of short RNA sequences or rare proteins that would otherwise be below the detection threshold [210,212]. This technique is vital for studying intratumoral transcriptome heterogeneity in gastric biopsy specimens.

Also, TSA signal amplification entails enzymatic deposition by HRP on tyramide (Tyr-HRP), in the presence of hydrogen peroxide. HRP conjugated to cell-bound detection antibodies catalyzes the oxidation reaction of tyramide, generating highly reactive, short-lived, phenol-type free radicals [213]. These oligomerize or deposit on local cellular macromolecules [213].

A critical aspect is optimizing the H_2_O_2_ concentration, as too much can inactivate the HRP enzyme or degrade the antigen, while too little limits free radical production [214]. Systems such as Alexa Fluor SuperBoost have refined these variables to provide an optimal signal-to-noise ratio.

##### mIHC and TME Characterization

The efficacy of modern therapies, especially immunological ones, depends not only on the presence of a receptor on cancer cells, but also on the composition and spatial organization of cells in TME [215,216,217,218]. mIHC allows the simultaneous visualization of multiple cell types over 23 markers on a single FFPE (formalin-fixed paraffin-embedded) slide, providing a systemic perspective at the microscopic level [219].

There are two main approaches in mIHC: chromogenic detection and mIF. Chromogenic detection is a colorimetric method based on HRP to convert colorless substrates (chromogens) into visible stains, usually used in IHC. mIF uses TSA to deposit stable fluorophores, allowing successive rounds of staining and antibody removal (stripping) without degrading the previous signal. An optimized stripping buffer based on SDS (sodium dodecyl sulfate) and Tris-HCl at pH 6.8 with β-mercaptoethanol ensures complete removal of primary and secondary antibodies, preventing false-positive results through cross-reactivity. The advantages of mIHC are summarized in Table 6 [215,216,217,218].

By mIHC, it has been shown that the response to immunotherapy in gastric cancer is more robust in patients with deep infiltration of cytotoxic T cells into tumor “nests” compared to those in whom immune cells are blocked in the stroma (the “immune-excluded” phenotype). Also, analysis of Natural Killer (NK) cell function and cyclooxygenase-2/prostaglandin E2 (COX-2/PGE2) expression by multiplexing revealed that high levels of PGE2 suppress NK lymphocyte activity [215,216,217,218], favoring tumor progression [220]. These details are invisible to traditional IHC methods and allow for a much finer selection of patients for combination therapies (e.g., anti-PD-1 plus anti-VEGF) [217].

##### Multiplexing Reagents and Translucent Chromogens

*The Brightfield Color Revolution*

A historical limitation of DAB was its opacity, which prevented visualization of two biomarkers at the same site (co-localization). The advent of translucent chromogens revolutionized brightfield IHC.

New generations of HRP-chromogens (e.g., Discovery Purple, Yellow, Green, Red, and Black) are designed with narrow absorption spectra. When two such dyes are deposited in the same cellular compartment, they produce a third color through optical mixing, allowing the pathologist to identify co-expression directly on the glass slide, without the need for fluorescence microscopy [221].

This capability is essential in gastric cancer for: (i) identifying dual positivity for HER2 and PD-L1 on the same tumor cells, a configuration that may suggest combined treatment strategies; (ii) differentiating between PD-L1-expressing tumor cells and nearby immune cells, critical for accurate calculation of the CPSs; and (iii) simultaneous assessment of proliferation markers (Ki-67) and therapeutic targets (CLDN18.2) to understand the aggressiveness of positive subclones [92,222].

*Nanotechnology and Nanozymes: Redefining Catalysis in IHC*

The integration of nanomaterials, such as *quantum dots* (*QDs*) as visualization markers, in GC diagnostics is one of the most advanced areas of current research. QDs are semiconductor nanocrystals that offer significant advantages over organic dyes and DAB in certain applications. They exhibit intense fluorescence, high resistance to photobleaching, and a tunable emission spectrum depending on the particle size [223].

In GC studies, QDs-IHC have been used to detect autophagy markers such as microtubule-associated protein 1 light chain 3B (LC3B) and stromal proteins, such as caveolin-1 (Cav-1), with much higher quantitative accuracy than DAB. Furthermore, QDs allow for spectral multiplexing, where multiple signals can be digitally separated even if they partially overlap, facilitating the TME complexity study [224,225,226].

Nanozymes based on molybdenum disulfide (MoS2) and gold nanoparticles (AuNP) are nanomaterials that possess intrinsic catalytic activity similar to peroxidase. MoS2/AuNP nanozymes have been used to construct ultrasensitive biosensors for gastric cancer [227], due to the fact that these can catalyze the DAB reaction with superior efficiency to natural HRP, as they are more stable to temperature and pH variations.

Nanozymes also enable stable, tunable, and multifunctional cancer therapies by exploiting the TME [228]. An integrated system using MoS2 and HRP (triple amplification) was able to detect miR-19b-3p (a small, non-coding RNA molecule and a key member of the oncogenic miR-17–92 cluster) acting as a regulator of gene expression at a concentration of 0.7 aM (Attomolar = 10^−18^ moles), offering enormous potential for early non-invasive diagnosis [229].

##### Digital Pathology and Quantitative Image Analysis

The transition from analog examination under a microscope to digital image analysis (DIA) in whole-slide imaging (WSI) represents a major qualitative leap in the standardization of gastric pathology [93,230]. DIA eliminates interobserver variation by applying rigorous mathematical thresholds for staining intensity and cellular morphology. Elite software platforms such as HALO, Visiopharm, and QuPath are capable of processing millions of cells in minutes, providing precise metrics [230,231,232] that are impossible to obtain manually, which are systematized in Table 7.

#### 4.5.3. Artificial Intelligence (AI) and Deep Learning Architectures

Artificial intelligence is no longer just an assistance tool, but is becoming a central component of precision diagnosis, capable of mitigating the limitations of IHC by “learning” complex models from large datasets [93,233,234,235,236].

In GC histopathological analysis, convolutional neural networks (CNNs) are the most widely used due to their superior ability to extract spatial features [237,238,239]. Models such as ResNet-50, VGG-16, and InceptionV3 have been trained on thousands of digital images to detect malignancy with an accuracy that often exceeds 97% [93].

A major innovation is the use of “fusion models”, which combine the strengths of multiple architectures (e.g., CNNs for local features and Transformers for the global blade context) [238,239]. These models are trained through an intermediate fusion process, where feature maps are concatenated before the final prediction, ensuring greater resilience to color variability [239].

#### 4.5.4. AI-Assisted Interpretation of PD-L1 and HER2

For PD-L1 CPS scoring, AI systems have demonstrated high agreement with expert consensus (Kappa coefficient of 0.78), providing crucial standardization for immunotherapy decisions [233]. Algorithms such as RepVGG have also been validated for HER2 scoring, achieving 94.0% accuracy, which helps eliminate ambiguities in IHC 2+ cases [235,240]. The explainability of these models (“explainable AI”—XAI) is essential for clinical adoption. Techniques such as Grad-CAM and LIME generate heatmaps that visually indicate to the pathologist the areas considered by the algorithm to make a diagnosis (e.g., nuclear atypia or glandular irregularities), increasing the physician’s confidence in the system [236,238,239].

### 4.6. Automation and Quality Control in the Modern Laboratory

Human errors and inter-laboratory variability are significantly reduced by fully automating processing [183]. Systems such as BenchMark ULTRA, Autostainer Link 48, and Leica BOND RX handle every step from deparaffinization to staining with chromogen or fluorophore [183].

A notable advance is the use of “PT Link”, an integrated pre-treatment module that combines deparaffinization, hydration, and antigen unmasking in a single step [183].

However, studies indicate technical risks, such as deformation of plastic racks at high temperatures of 97 °C, which can lead to tilting of slides and the appearance of “rail artifacts” (uneven staining at the edges of the slide) [183]. The optimal solution identified is the use of dedicated racks for the unmasking phase, different from those used in the staining apparatus [183].

The use of genetically modified cell lines in liquid suspension (CLF) form represents a superior alternative to tissue fragments for quality control [241]. These are automatically dropped onto the slide by the IHC system (e.g., LYNX480 PLUS), ensuring a perfectly homogeneous cell distribution and precise monitoring of antibody and reagent performance in each run [241].

### 4.7. Sustainability and Green Chemistry in the Pathology Laboratory

In the period 2025–2026, diagnostic laboratories are increasingly focusing on reducing their environmental impact [242]. Although DAB is an essential reagent, its potentially carcinogenic nature requires safety and waste management measures. Innovations in this area include: stable formulations, whereby DAB reagents that remain active for 14 days significantly reduce the amount of chemical waste generated by the disposal of daily preparations; recirculation systems; modern automation allows for the selective collection of toxic chromogens, preventing wastewater contamination; eco-friendly materials; and the transition from single-use plastics to biodegradable or durable borosilicate glass consumables in tissue processing steps [243,244].

Although IHC on tissue sections remains the standard, progress in diagnostic reagents is also reflected in in vivo and intraoperative imaging techniques.

**Figure 6:**
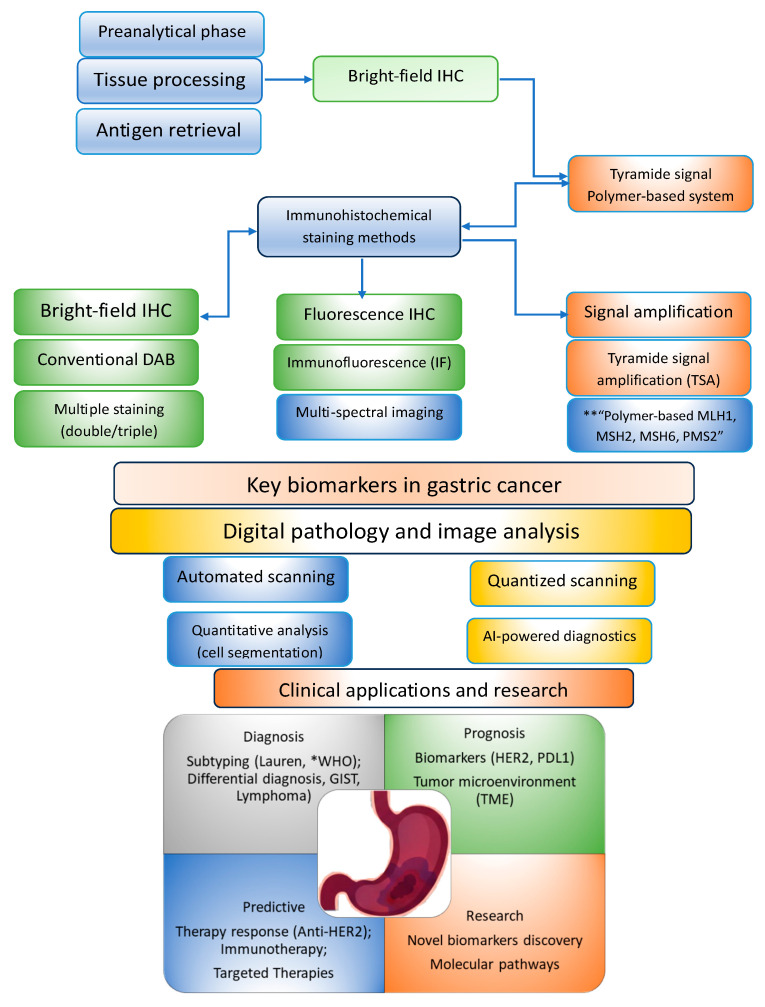
Advanced techniques in gastric cancer immunohistochemistry and their clinical impact. * WHO—World Health Organization. “The 5th edition (2019) WHO classification of stomach tumors focuses on histological appearance, categorizing them into tubular, papillary, mucinous, poorly cohesive, and rare variants”. ** “polymer-based MLH1 MSH6 PMS2” refers to a sensor based on graphene and cobalt-protoporphyrin complex used for the recognition and quantification of biomarkers, such as MLH1, MSH2, MSH6, and PMS2.

The summary sequence of advanced IHC techniques corroborated their impact on clinical applications targeting diagnostic, prognostic, predictive, and research perspectives is presented in Figure 6.

## 5. Perspectives

Perspectives for the coming years include the following aspects:

1. Standardization of “Reflex Testing”: Implementation of automated protocols where each gastric biopsy is immediately tested for HER2, MSI, PD-L1, and CLDN18.2, shortening the time until personalized treatment.
2. Transition to all-digital diagnostics: Laboratories can adopt routine scanning of DAB-stained slides, using AI algorithms to identify focal heterogeneity that might be missed during manual examination.
3. Development of environmentally friendly reagents: Systematic replacement of toxic components in IHC kits with biodegradable alternatives, without compromising diagnostic sensitivity.
4. Brightfield multiplexing: Widespread adoption of translucent chromogens to replace costly immunofluorescence in routine diagnostics.
5. The future of gastric diagnostics may be defined by:
  - Spatial “Omics” integration: Combining mIHC with spatial transcriptomics (e.g., Visium) to simultaneously map protein and gene expression profiles in specific cellular niches [218,245,246,247].
  - Foundational AI models: Developing algorithms trained on gigantic multi-center datasets that can predict not only the status of a biomarker, but the entire evolutionary trajectory of the disease and the risk of relapse under treatment [39,93,234,237,248].
  - Digitalized quality control: Replacing classic tissue controls with “virtual controls” and automatically monitoring staining intensity through DIA algorithms to ensure global diagnostic uniformity [241].

These technological advances are not simple laboratory refinements, but are vital tools that transform gastric cancer from a disease with a reserved prognosis to a manageable condition through individually optimized surgical, chemotherapeutic, and immunological interventions.

Ultimately, the integration of these technological advances ensures that the pathologist remains at the center of clinical decision-making in gastric cancer, providing the oncologist with the molecular appearance needed to get to the essence of the biological complexity of this devastating neoplasm. The continued refinement of IHC reagents and imaging methods holds the promise of longer survival and improved quality of life for patients worldwide.

## 6. Conclusions

Immunohistochemistry has redefined the way gastric malignancies are detected, classified, and treated. Its undeniable advantages—low cost, morphology preservation, rapidity, and the ability to deliver direct therapeutic targets (HER2, PD-L1, and Claudin 18.2)—keep it at the center of oncological pathology.

The landscape of immunohistochemistry in gastric cancer for 2026 is one of technological integration and molecular refinement.

From the precise detection of Claudin 18.2 to the use of artificial intelligence for prognostic prediction, gastric pathology has become a field of high precision.

Therapeutic success now depends on the ability of laboratories to deliver rapid, reproducible, and deeply detailed results, transforming each biopsy fragment into a strategic map for the patient’s life.

Technical standardization, adoption of digital pathology, and multidisciplinary vision are the keys that will define success in the fight against this aggressive neoplasm in the near future.

The study of advances in the use of DAB and diagnostic reagents in gastric cancer reveals a discipline that is rapidly maturing from descriptive staining to high-resolution quantitative analysis. DAB maintains its central role due to its stability and versatility, but is now part of a much more complex ecosystem.

Polymer amplification systems and TSA have pushed the limits of detection to previously inaccessible molecular levels, enabling the success of therapies such as anti-CLDN18.2 or anti-FGFR2b.

At the same time, nanotechnology and artificial intelligence provide the tools necessary to process the huge volume of information generated by new multiplexing protocols. However, the success of IHC is entirely dependent on the standardization of processes.

The disadvantages related to pre-analytical variability (fixation) and subjectivity of interpretation emphasize the need for continuous staff training and the adoption of digital assistance technologies. As the arsenal of targeted therapies expands, IHC will continue to evolve, moving from a qualitative “presence/absence” method to a highly accurate digital quantification method.

## Figures and Tables

**Table 1:** Stages of the Correa cascade, morphological characteristics, and relevant IHC markers.

Correa Cascade Stage	Morphological Features	Relevant IHC Markers
Chronic gastritis	Lymphoplasmacytic inflammatory infiltrate	Markers of inflammation
Gastric atrophy	Loss of native gastric glands	MUC5AC, MUC6
Intestinal metaplasia	Replacement with intestinal-type epithelium	CDX2, MUC2, CD10
Dysplasia (LGD */HGD **)	Cytological and architectural atypia	Ki-67, p53
Adenocarcinoma	Basement membrane invasion	Pancytokeratin, HER2

* LGD: low-grade; ** HGD: high-grade.

**Table 2:** Mechanism, diagnostic context, and management implications of risk factors leading to gastric cancer.

Risk Factor	Mechanism and Diagnostic Context	Management Implications	Ref.
*Helicobacter pylori*	Induces chronic inflammation and the precancerous Correa cascade	Eradication is a priority; it requires reflex testing at diagnosis	[59,70,72]
Epstein–Barr virus	Promoter hypermethylation and PD-L1 upregulation	Identifies a subtype sensitive to immunotherapy (10% of cases)	[71,73]
Genetic Predisposition	Germline mutations in CDH1, Lynch, and FAP * genes	Requires family screening and, in some cases, prophylactic gastrectomy	[74]
Environmental Factors	High-salt diet, smoking, and central obesity	Influence anatomical location (cardia vs. non-cardia)	[75]
Atrophic Gastritis	Parietal cell loss and hypochlorhydria	High risk indicator; requires rigorous endoscopic monitoring	[76,77]

* FAP—Familial adenomatous polyposis.

**Table 3:** Tumor types, key IHC markers, and concise interpretation.

Suspected Tumor Type	Key IHC Markers	Interpretation	Ref.
Adenocarcinoma	CK7, CK20, CDX-2, MUC2	Variable CK7+/CK20+ profile; CDX-2 confirms intestinal differentiation	[83]
Gastric lymphoma (e.g., Hodgkin)	CD15, CD30, PAX5, MUM1	CD30 and CD15 confirm Reed–Sternberg cells in PGHL *	[84,85]
Gastrointestinal stromal tumor (GIST)	CD117 (c-kit), DOG1, CD34	CD117 positivity is defining for most GISTs	[86,87]
Neuroendocrine tumor	SPY **, CgA ***, Ki-67	Positivity of neuroendocrine markers confirms the origin	[88,89,90]

* PGHL usually refers to Primary Gastric Hodgkin Lymphoma; ** SPY: Synaptophysin—integral glycoprotein of the synaptic vesicle membrane localized in presynaptic neurons and neuroendocrine cells; *** CgA: acidic protein localized in the secretory granules of neuroendocrine cells that releases peptide hormones. SPY and CgA are the primary immunohistochemical markers used to identify neuroendocrine cells and tumors.

**Table 4:** The algorithm of HER2 testing.

HER2 IHC Score/ Interpretation	Biopsy Criteria *	Resection Criteria **	Clinical Decision
0 (Negative)	No staining or membrane staining in <5 cells	No staining or membrane staining in ≤10% of cells	Not eligible for anti-HER2 therapy
1+ (Negative)	Weak membrane staining, visible only at high magnification	Weak/incomplete membrane staining in ≥10% of cells	Not eligible for anti-HER2 therapy
2+ (Equivocal)	Weak/moderate membrane staining, visible at medium magnification	Weak/moderate membrane staining in ≥10% of cells	Requires reflex FISH testing
3+ (Positive)	Strong membrane staining, visible at low magnification	Strong/complete or basolateral membrane staining in ≥10% of cells	Eligible for Trastuzumab

* Cluster of ≥5 cancer cells; ** Sentinel lymph node biopsy, SLN ≥ 10%.

**Table 5:** Antibody clones, platforms and detection, positivity criteria, and therapeutic impact of precision biomarkers in gastric cancer.

Biomarker	Antibody Clone	Platform/ Detection	Positivity Criteria	Therapeutic Impact	Ref.
HER2	4B/SP3	Ventana UltraView/DAB	IHC 3+	Trastuzumab blocks tumor growth signals and stimulates the immune system to destroy cancer cells	[180,181]
PD-L1	22C3/28-8	Dako Link 48/DAB	CPS ≥ 1 or CPS ≥ 5	Pembrolizumab stimulates the immune system to destroy tumor cells, and Nivolumab activates the body’s own immune system to attack tumors	[182,183,184,185]
CLDN18.2	43-14A	43-14A	≥75% cells (2+/3+)	Zolbetuximab, * marketed as Vyloy, is a first-in-class monoclonal antibody approved in 2024 for treating advanced HER2-negative gastric cancer or GEJ ** adenocarcinomas that are Claudin (CLDN) 18.2-positive	[170,186,187,188,189]
FGFR2b	FPR2-D	Polymer/DAB	≥10% cells (2+/3+)	Bemarituzumab blocks fibroblast growth factors and inhibits pro-tumor signaling in gastric and GEJ ** cancers that overexpress FGFR2b	[178,190,191,192]

*“2024- FDA Approval: The U.S. Food and Drug Administration (FDA) approved zolbetuximab (Vyloy) in combination with chemotherapy for HER2-negative, CLDN18.2-positive gastric or gastroesophageal junction adenocarcinoma, based on the results of the phase III SPOTLIGHT and GLOW trials” [189]; ** GEJ—Gastroesophageal junction.

**Table 6:** mIHC advantages and its benefits in gastric cancer [215,216,217,218].

mIHC Advantage	Technical Explanation	Benefits in Gastric Cancer
Tissue saving	Analysis of dozens of markers on a single 4 μm section	Vital for small and precious endoscopic biopsies
Proximity analysis	Measurement of the distance between CD8+ cells and the tumor	More accurate in predicting the anti-PD-1 response compared to simple PD-L1 IHC
Identification of tertiary lymphoid structures (TLS)	Co-localization of * B, ** T, and *** DCs markers	Identification of tertiary lymphoid structures correlated with a favorable prognosis
Exhaustive phenotyping	Distinction between M1 (pro-inflammatory) and M2 (immunosuppressive) macrophages	Understanding mechanisms of resistance to immunotherapy in the TME

* B cells: CD19, CD20, CD22, CD27, and CD138; ** T cells: CD3, CD4, and CD8 (Cytotoxic); *** DCs—Dendritic cells: CD11c, MHCII, CD83, CD1c, CD141, CD123, and CD207.

**Table 7:** Main targeted metrics, applications in gastric cancer, and impact on diagnosis [230,231,232].

Metrics Analyzed	Application in Gastric Cancer	Diagnostic/Prognostic Impact
Optical Density (OD)	Accurate measurement of HER2 membrane staining intensity.	Objective distinction between 1+ and 2+ scores, reducing the need for reflex FISH tests.
Compartment Segmentation	Automatic separation of tumor area from necrotic or inflammatory stroma.	Calculation of PD-L1 CPS on strictly delimited tumor areas, eliminating necrotic “debris”.
Automatic H-Score	Integration of intensity (0, 1+, 2+, and 3+) with percentage of positive cells.	Provides a continuous numerical score for biomarkers, allowing fine statistical correlations with survival.
Nearest Neighbor Analysis	Calculation of average distance from tumor cell to nearest immune cell.	Identification of immune “hotspots” correlated with response to checkpoint inhibitors.

## Data Availability

The original contributions presented in this study are included in the article. Further inquiries can be directed to the corresponding author(s).

## Abbreviations

The following abbreviations are used in this manuscript:

IHC	Immunohistochemistry
GC	Gastric cancer
HER2	Human epidermal growth factor receptor 2
PD-L1	Programmed death-ligand 1
MMR	Mismatch repair
dMMR	Mismatch repair deficiency
MSI	Microsatellite instability
AI	Artificial intelligence
CLDN 18.2	Claudin 18.2
TCGA	The Cancer Genome Atlas
ACRG	Asian Cancer Research Group
DAB	3,3′-diaminobenzidine
CPS	Combined positive score
PCR	Polymerase chain reaction
mAbs	Monoclonal antibodies
pAbs	Polyclonal antibodies
RT-PCR	Reverse transcription-polymerase chain reaction
EBV	Epstein–Barr virus
CIN	Chromosomal instability
EOGC	Early-onset gastric cancer
FAP	Familial adenomatous polyposis
WLE	White light endoscopy
GIST	Gastrointestinal Stromal Tumor
PGHL	Primary Gastric Hodgkin Lymphoma
SPY	Synaptophysin
CgA	Acidic protein
HRP	Horseradish peroxidase
PCNA	Proliferating cell nuclear antigen
VEGF	Vascular endothelial growth factor
ToGA	Trastuzumab for Gastric Cancer
ADCs	Antibody–drug conjugates
FISH	Fluorescence in situ hybridization
ISH	In situ hybridization
CISH	Chromogenic in situ hybridization
IICs	Infiltrating immune cells
ICIs	Immune checkpoint inhibitors
LELGC	Lymphoepithelioma-like gastric carcinoma
NGS	Next-generation sequencing
ASCO/CAP	American Society of Clinical Oncology/College of American Pathologists
FGFR2b	Fibroblast growth factor receptor 2b
GEJ	Gastroesophageal junction
CDX2	Caudal-type homeobox 2
SATB2	Special AT-rich sequence-binding protein 2
GAED	Gastric Adenocarcinoma with enteroblastic differentiation
GPC3	Glypican-3
SALL4	Spalt-like family of 4 C_2_H_2_ zinc finger transcription factors
AFP	Alpha-fetoprotein
GA-FG	Gastric adenocarcinoma of the fundic gland
EMT	Epithelial–mesenchymal transition
TSA	Tyramide signal amplification
HRC	Hybridization chain reaction
mIHC	Multiplex immunohistochemistry
mIF	Multiplex immunofluorescence
TME	Tumor microenvironment
CD8+	T lymphocytes or cytotoxic T cells
CTCs	Circulating tumor cells
TLS	Tertiary lymphoid structures
NK	Natural killer cell function
LC3B	Microtubule-associated protein 1 light chain 3B
Cav-1	Caveolin-1
COX-2/PGE2	Cyclooxygenase-2/prostaglandin E2
QDs	Quantum dots
DIA	Digital image analysis
WSI	Whole-slide imaging
CNN	Convolutional neural network
